# Vulvar Cancer: Facing a Rare Disease

**DOI:** 10.3390/cancers14061581

**Published:** 2022-03-20

**Authors:** Mario Preti, Denis Querleu

**Affiliations:** 1 Department of Surgical Sciences, University of Torino, 10124 Torino, Italy; 2 Division of Gynecologic Oncology, Fondazione Policlinico Universitario A Gemelli IRCCS, 00168 Roma, Italy; denis.querleu@esgo.org; 3 Department of Obstetrics and Gynecologic Oncology, University Hospital of Strasbourg, 67000 Strasbourg, France

“We must never be afraid to go too far, for truth lies beyond.”

Marcel Proust, *In Search of Lost Time*

When we proposed the topics for this Special Issue, our first thought was to integrate the results from the latest original research to practical reviews focusing on key questions. Both authors of this editorial are former presidents of international societies, Mario Preti from the International Society for the Study of Vulvovaginal Disease (ISSVD, www.issvd.org (accessed on 8 March 2022)), Denis Querleu from the European Society of Gynaecological Oncology (ESGO, www.esgo.org (accessed on 8 March 2022)). The ESGO evidence-based guidelines cover the whole field of gynecologic oncology. The ESGO guidelines for the management of vulvar cancer were published in 2017 [1] with particular attention to improve the quality of care for the management of nodal disease [2] and the rational use of (chemo)radiation. The aims of the ISSVD to promote international communication among gynecologists, pathologists, dermatologists, and other healthcare providers [3] have been recently highlighted by a series of position papers wherein the role of scientific societies and committees are underlined [4]. Both societies are working to bridge the remaining gap between clinical research and affected women.

The striking fact is that advanced vulvar cancers have not disappeared today, in the first quarter of the 21st century, and that early cases are still managed in non-specialized centers. To our knowledge, only the Danish government has taken the initiative to centralize the management of vulvar cancers in only two centers in the country. On the other hand, vulvar carcinoma is a rare cancer [5,6] and should be added to the list of rare diseases, then benefiting from the corresponding special interest. It is estimated that one in four patients with cancer in Europe has a rare cancer, leading to the need for specific strategies in 25% of the total cancer burden [7]. How many articles begin with the sentence “Vulvar cancer is a rare cancer and affects about 4 in 100,000 women/year”? This sentence inspires the feeling that a rare cancer is a double adversity; adding the burden of the neoplasm leads to a feeling that the prevention does not work, that not enough attention has been given to a disease that could have been diagnosed at an earlier stage, or, even better, at the level of intraepithelial lesion.

More than 20 years ago, an editorial in *The Lancet* stated that rare cancers are often inadequately diagnosed and treated [8]. This justifies the focus of this Special Issue on vulvar cancer oncogenesis, its clinical and histopathological diagnosis, multimodal therapeutic approach, adjuvant treatment, and tailored follow-up, encompassing the need for psycho-sexuological support.

It is clear that the therapeutic approach has much improved, but we must not forget that in some countries there are different possibilities of access to treatment and prevention. The epidemiologic characteristics of vulvar squamous cell cancer (VSCC) represent an impediment to the adoption of screening strategies, so VSCC detection often occurs as an incidental finding during routine gynecologic visits. Indeed, VSCC prevention and early diagnosis also show a weak side in high-standard health services, even though scheduled cytologic or human papillomavirus (HPV) testing for cervical cancer screening could be an opportunity to perform a careful clinical inspection of the vulvar region, and to detect and treat multicentric HPV-related intraepithelial neoplasia. Even though only a small percentage of VSCCs are HPV-related [9], a recent meta analysis has shown that the risk of vulvar cancer after cervical intraepithelial neoplasia treatment is significantly increased in treated patients compared with the general population (RR 3.34, CI 2.39–4.67; *p* < 0.001) [10]. The risk is higher in women over 50, and remains elevated for 20 years after the treatment of the cervical disease [10,11]. This suggests the need for ad hoc prolonged prevention strategies in this at-risk group, in order to promote early detection of asymptomatic vulvar cancer, as well as other HPV-related neoplasm post-cervical intraepithelial neoplasia treatment.

On the other hand, lichen sclerosus is not a rare disease [12]. There is a need to draw the attention of affected women to make them aware of the risk of developing non-HPV-related vulvar neoplasms [13,14]. Concentrating our diagnostic efforts in the context of lichen sclerosus is a way to reduce the long-term risk of invasive carcinoma and to provide an evidence- and risk-based profile for affected patients [12,15].

Moving forward, what are our goals and the direction to dedicate expanded effort and resources [16,17]? We are aware that a reduction in the incidence and mortality of vulvar cancer requires a huge organizational effort, in particular for a rare cancer such as vulvar cancer. There is a need to implement infrastructure to accommodate this. The filter activity that is performed at primary/secondary care levels has demonstrated to be insufficient, and there is a need to enforce referral pathways to hub centers for vulvar disease. Implementing specialist multidisciplinary clinics for VSCC and precursor lesions is a public health priority [18]. We also need to foster responsibility in teaching and education, with public health programs for women’s cancer prevention and detection, involving strengthened partnerships with patients’ associations to extend our multidisciplinary specialty. Finally, performing standard clinical trials with innovative study designs improving procedures for VSCC early diagnosis and treatment is badly needed.

This Special Issue is composed of contributions from authors coming from diverse regions. All have extensive experience in their fields. They are international authorities and have submitted papers that provide cutting-edge evidence on multiple aspects of vulvar cancer. We wholeheartedly thank them for dedicating their time and sharing their expertise.
